# Integrated 3D-Printed Microfluidic Device for Immunocapture and Electrochemical Assessment of Transferrin Saturation in *Point-of-Care* Stroke Diagnostics

**DOI:** 10.1021/acssensors.5c02834

**Published:** 2025-12-26

**Authors:** Davide Paolini, Silvia Dortez, Marta Pacheco, Teresa Gasull, Dario Compagnone, Flavio Della Pelle, Alberto Escarpa

**Affiliations:** † Department of Analytical Chemistry, Physical Chemistry and Chemical Engineering, University of Alcala, 28802 Alcala de Henares, Madrid, Spain; ‡ Department of Bioscience and Technology for Food, Agriculture and Environment, 19031 University of Teramo, Campus “Aurelio Saliceti” Via R. Balzarini 1, 64100 Teramo, Italy; § Cellular and Molecular Neurobiology Research Group, Department of Neurosciences, Germans Trias i Pujol Research Institute (IGTP), 08916 Badalona, Barcelona, Spain; ∥ Chemical Research Institute “Andrés M. Del Río” (IQAR), University of Alcala, 28802 Alcala de Henares, Madrid, Spain

**Keywords:** fused deposition modeling, 3D-sensor, transferrin, transferrin-bound iron, square wave voltammetry

## Abstract

A 3D-printed electrochemical microfluidic device (3D-EMD) was developed to assess the transferrin saturation (TSAT) biomarker in ischemic stroke patients. The all-in-one 3D-EMD integrates a strategically engineered immunoassay module for the direct and selective isolation of transferrin (Tf) from unpretreated clinical samples, unaffected by sample coloration, with an interchangeable electrochemical sensor for the simultaneous detection of Tf and Tf-bound iron. Both modules are interconnected through microfluidic channels whose flow is regulated by a cylindrical rotary valve. The analytical workflow enables magnetic bead-based direct Tf immunocapture and simultaneous electrochemical detection of Tf and Tf-bound iron via square wave voltammetry, allowing TSAT assessment within 60 min using only 50 μL of sample. Validation with certified reference materials demonstrated excellent accuracy (*E*_r_ ≤ 5%) and precision (RSD ≤ 4%). Application to human serum from ischemic stroke patients showed strong correlation (*r* = 0.87) and agreement (slope 0.9 ± 0.3; intercept 6 ± 10; *p* < 0.05) with the urea-PAGE reference method, which typically requires up to 18 h. Overall, the 3D-EMD constitutes an elegant, fully integrated dual-functionality platform that seamlessly combines customizable sample preparation with online electrochemical detection in a single device. This configuration enables direct serum analysis and supports clinical decision-making in time-critical conditions. The device shows strong potential as a rapid *point-of-care* testing candidate for ischemic stroke and as a next-generation platform for broader clinical diagnostics.

## Graphical abstract

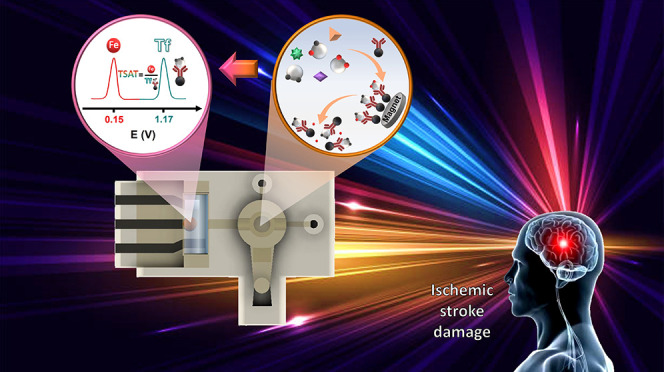

Microfluidics is the art of manipulating small volumes of fluid in micrometric-scale channels/systems.^1^ This discipline offered captivating solutions in scientific and industrial applications,^2^ allowing overcoming several analytical issues related to the traditional macro-scale fluidic counterpart. The advantages include full miniaturized integration of (bio)­analytical steps, reduced consumption of samples and reagents, faster analysis time, increased portability, and enhanced sensitivity and reproducibility.^3^ Furthermore, the scalability of microfluidic manufacturing enables the development of highly automated and integrated systems, making microfluidic technology an attractive solution for a plethora of applications in diagnostics, and chemical analysis.^4,5^

Electrochemical detection techniques are highly valued for their sensitivity, selectivity, speed, accuracy, simplicity, and low cost. These methods require minimal sample amounts and are easily integrated into compact systems, including emerging smartphone-based and wearable devices.^6–8^ In particular, the coupling of microfluidic systems with integrated electrochemical detectors offers disruptive solutions combining small-fluid handling capabilities, analytical sensitivity, and the ability to automate/integrate different analysis steps.^9,10^ Properly conceived electrochemical microfluidic devices (EMDs) can offer several benefits, including real-time monitoring of dynamic processes, low power consumption, the ability to work directly with unpretreated samples, and the possibility of multiplexed analysis in compact cost-effective setups.^11^ For these reasons, EMDs have been used for the detection of a plethora of analytes,^12,13^ making them particularly appealing for *point-of-care* testing (POCT). Indeed, the incorporation of microfluidic technology into POCT devices brings transformative benefits to healthcare, such as portability, reduced reagent consumption, and fast response times, potentially improving diagnostic efficiency and accuracy, while enabling early disease detection, real-time therapeutic monitoring, and broader accessibility (especially in resource-limited settings).^14–16^

Recent advances in additive manufacturing, particularly three-dimensional (3D) printing, have significantly influenced the fabrication of EMDs and electrochemical cells.^17^ Traditionally, the manufacturing of such devices relied on complex lithographic techniques, which were both time-consuming and costly.^18^ In contrast, 3D printing technologies have emerged as a cost-effective, customizable, and scalable alternative, enabling the rapid development of highly functional EMDs, as well as the assembly of complex devices, by integration of rationally designed components.^19^ Furthermore, through the design flexibility and customization offered by 3D printing, it is possible to manufacture microfluidic platforms containing multiple channels or electrochemical cells within the same device,^20^ allowing for the multiplexed detection of several biomarkers or analytes of clinical interest. Among 3D printing technologies, fused deposition modeling (FDM) has gained a notable reputation since it is accessible and capable of fabricating intricate geometries using thermoplastic polymers.^21^ FDM facilitates rapid device prototyping with precise control over structural parameters;^22^ moreover, it enables the generation of well-defined (micro)­fluidic flows within the channels of miniaturized devices, even when their geometrical features do not reach the micrometer scale.^20,23,24^ Nevertheless, still some issues that need to be improved and implemented, in particular to design integrated microfluidic and electrochemical systems.^25,26^

To overcome the limitations inherent to conventional 3D-printed electrochemical platforms (particularly the limited performance of printed electrodes and the challenges in postfabrication surface modification/activation^23^) a hybrid fabrication methodology has been introduced.^24^ This approach integrates fused filament fabrication (FFF), using both conductive and nonconductive filaments, with ink printing, enabling direct modification of the electrode surface after the 3D printing process. Overall, the resulting 3D platforms show enhanced functionality and are well-suited for a wide range of applications, including electrochemical sensors, and lab-on-a-chip devices.^27,28^

Recently, different electrochemical and colorimetric microfluidic paper-based analytical devices have been proposed as POCTs for the reliable assessment of transferrin saturation (TSAT) in patients who have suffered ischemic strokes.^29,30^ TSAT is considered an important biomarker to assess the general body iron status, and it is used to diagnose iron deficiency and overload.^31,32^ In particular a high TSAT level is associated with an increased risk of stroke and brain damage induced by ischemia.^33,34^ TSAT (%) is defined as the ratio between the amount of serum iron (Tf-bound iron) and the total iron-binding capacity (TIBC), that is the maximum amount of iron that can be captured by transferrin (Tf).^32^ Despite the notable advantages offered by the proposed paper-based analytical devices, compared to the urea-PAGE method,^34,35^ they have certain limitations in the TSAT assessment. The most significant is the requirement for sample pretreatment to be carried out off-device; this process introduces additional complexity, potential contamination, and variability in results.^29,30^ Furthermore, the paper-based platforms had constraints in terms of mechanical stability and reproducibility, which limited their potential for widespread adoption in clinical settings.

In addition, in the context of TSAT assessment, specifically, in paper-based systems, colorimetric detection is commonly employed due to its simplicity and the favorable optical properties of paper, whose white background provides excellent contrast for visual detection. Electrochemical detection, in contrast, mainly relies on the versatility of the stencil-printed format. However, integrating immunoassays within paper-based platforms is extremely challenging, whereas 3D printing offers a clear advantage owing to the intrinsic flexibility of additive manufacturing technologies, which are inherently better suited for such complex integrations. In 3D-printed systems, the focus shifts toward the use and modification of conductive filaments as electrodes, thereby justifying an exclusive emphasis on electrochemical detection. Indeed, 3D-based electrochemical sensing constitutes a versatile strategy that synergistically combines the customizability and structural precision of 3D printing with the material adaptability of stencil or 3D-mask printing. This integration enables the incorporation of high-performance sensors directly onto 3D-printed conductive filaments and contacts, ultimately simplifying the detection process, which becomes entirely electrochemical.

In this study, an all-in-one 3D-printed electrochemical microfluidic device (3D-EMD) has been developed for the assessment of TSAT in untreated human serum samples. 3D-EMD consists of an on-valve immunoassay module for the selective capture of Tf and an interchangeable complete electrochemical three-electrodes cell for simultaneous and sensitive electrochemical detection of Tf and Tf-bound iron communicating through microfluidic channels whose flow is regulated by a cylindrical rotary valve. The electrochemical cell was fabricated using FDM, using conductive and nonconductive filaments, boosting the sensing capabilities with gold nanoparticles (AuNPs). In the following sections, we will demonstrate that the proposed approach enables the direct, fast, and reliable TSAT assessment in serum samples obtained from ischemic stroke patients.

## Materials and Methods

### Reagents and Samples

Iron­(III) chloride hexahydrate, tetrachloroauric acid, hydroxylamine hydrochloride, human transferrin (T3309), sodium hydroxide, acetic acid glacial, sodium acetate anhydrous, potassium hexacyanoferrate (II) trihydrate, and citric acid were purchased from Merck (Darmstadt, Germany). Ortho-phosphoric acid, potassium chloride, disodium hydrogen phosphate, and sodium chloride were purchased from Scharlau (Barcelona, Spain). Sodium dihydrogen phosphate and potassium hexacyanoferrate (III) were purchased from PanReac (Barcelona, Spain). Boric acid was acquired from Fluka (Darmstadt, Germany). Human serum (certified reference material, Spintrol H normal 1002121) was purchased from Spinreact (Girona, Spain). Anti-transferrin antibody (ab66952) was purchased from Abcam (Cambridge, UK). Fe^3+^ and Tf standard solutions were prepared daily by dissolving appropriate amounts in 0.2 M Britton–Robinson buffer solution pH 3.0 (BR). All reagents and solvents were of analytical grade. All solutions were prepared in Milli-Q water (Merck Millipore, Darmstadt, Germany).

Pseudonymized serum samples of the multicenter, randomized, double-blind, placebo-controlled TANDEM-1 (thrombolysis and deferoxamine in middle cerebral artery occlusion) study were used to evaluate TSAT by 3D-EMD and compare it with the urea-PAGE method.^34,35^ The serum samples were collected at the Hospital Universitari Germans Trias i Pujol (HUGTP) in untreated patients. All samples were stored at −20 °C before use. TANDEM-1 study was approved by the Spanish Drug Agency (eudraCT 2007-0006731-31) and by local Ethics Committees, including the HUGTP Ethics Committee, and was registered on https://clinicalTrials.gov as NCT00777140.

### Materials

A nonconductive natural polyethylene terephthalate glycol (PETG) filament (SmartMaterials, Spain) and a carbon black (CB) filled polylactic acid (PLA) filament (Protopasta CDP11705, Protoplant, Canada) were used to construct the electrochemical fluidic devices. A cleaning filament (SmartMaterials, Spain) was employed to remove impurities from the PLA-CB filament when exchanging filaments. Transparent PLA filament from Shenzhen Eryone Technology Co. (China) was used to integrate the immunostep in the device. Carbon paste (BG04) was purchased from SunChemical (New Jersey, USA). Silver/silver chloride (60/40) paste was purchased from Merck (Darmstadt, Germany).

AuNPs were synthesized using the Turkevich method.^36^ In brief, an aqueous solution of HAuCl_4_ (1 mM, 50 mL) was heated until boiling under constant stirring. Then, a sodium citrate solution (37 mM, 5 mL) was rapidly added, leading to a color change from pale yellow to deep red, indicating nanoparticle formation. The reaction mixture was then left to stir for 20 min. After cooling to room temperature, a preconcentration treatment was applied to the AuNPs dispersion to adjust it to 90 nM. 1 mL of AuNPs was centrifuged at 6.7 xg for 10 min. Then, 900 μL of the supernatant was discarded, and the remaining AuNP dispersion was placed in an ultrasonic bath for 5 min. The obtained AuNP colloid was used to modify the 3D-printed working electrode (see the *3D-EMD Design, Manufacturing, and Assay rotocol* section). Graphene quantum dots (GQDs) were synthesized using a straightforward aqueous-phase method.^37^ Initially, 1.6 M citric acid was heated at 150 °C under continuous stirring until a dark color was obtained. Subsequently, a solution of 50 mL of sodium hydroxide solution (0.1 M) was added dropwise to the mixture, leading to the formation of GQDs. AuNPs and GQDs were stored in a fridge before their use.

Pierce^TM^ Protein G Magnetic Beads of 1 μm particle size were acquired from Thermo Fisher Scientific (Rockford, USA). Anti-transferrin immunomagnetic beads (Anti-Tf-MBs) were used for the isolation of Tf from human serum samples. They were prepared according to the literature,^38^ as follows: 50 μL of 7.46 μg mL^–1^ anti-Tf antibody in PBS were added to 5 μL of MBs (10 mg mL^–1^) and incubated for 45 min at 25 °C, with agitation. The vial containing the anti-Tf-MB complex was placed on a magnet holding block for 2 min, and the supernatant was removed. Finally, two washing steps were performed with 100 μL of PBS (0.1 M, pH 7.4).

### Apparatus

The design of the 3D device was obtained using computer-aided design software (Fusion 360, Autodesk, student version) and printed by Prusa i3 MK3S+ equipped with a 0.4 mm nozzle and Original Prusa MK4S (Prusa Research, Czech Republic) equipped with a 0.2 mm nozzle. Electrochemical measurements were performed with a Palmsens 4 Potentiostat/Galvanostat/Impedance Analyzer (Palm Instruments BV, Houten, Netherlands) managed by PS Trace software. A microplate reader (Synergy HTX, BioTek) ultraviolet–visible (UV–vis) spectrometer, was used to study the AuNP colloid.

### 3D-EMD Design, Manufacturing, and Assay Protocol

Figure 1 illustrates the 3D-EMD design. The device design comprises an immunoassay module and an interchangeable 3D-printed electrochemical cell integrating a three-electrode system (please for further details see Figures S1 and S2).

**Figure 1:**
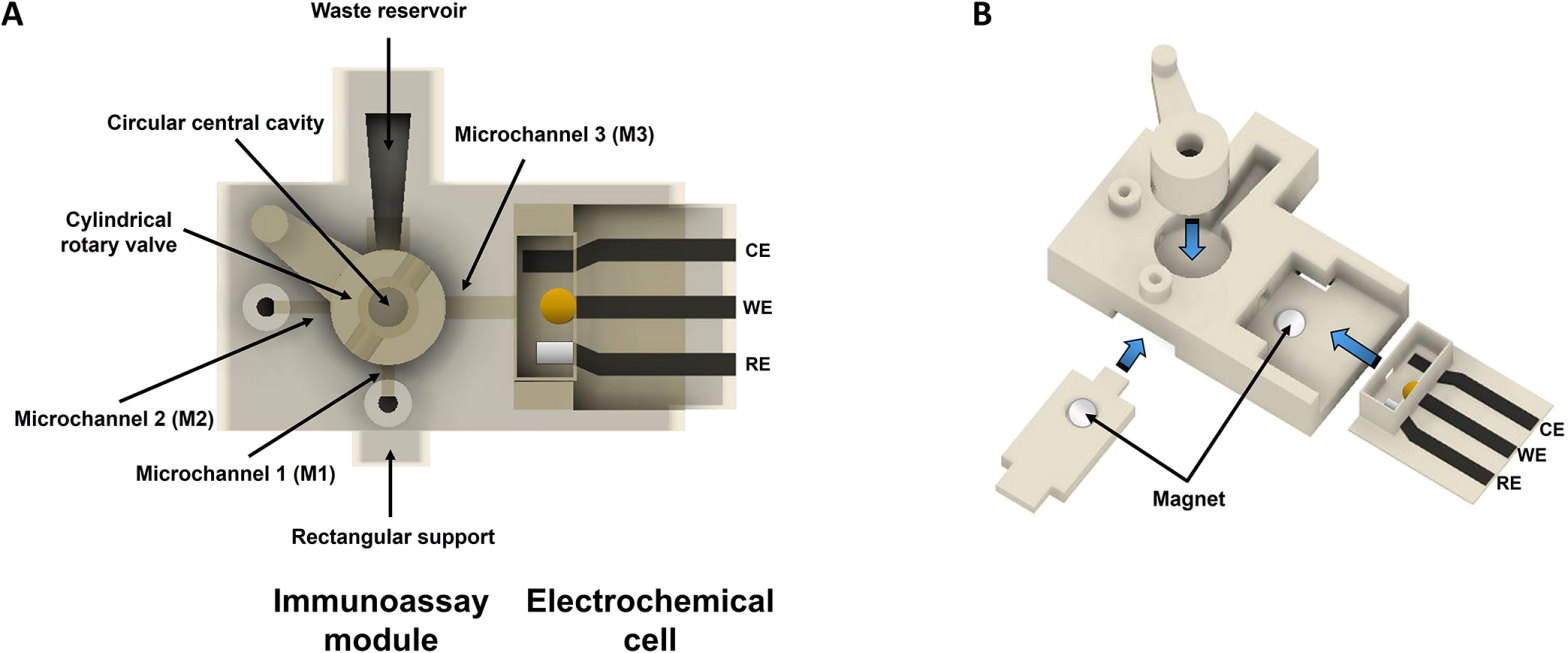
(A) 3D-EMD design integrating immunoassay module and complete 3D-based electrochemical cell. (B) Exploded view of magnet and rotatory valve holders as well as interchangeable 3D-based electrochemical cell.

The immunoassay module is shown in Figure 1A which consisted of a rectangular 3D structure composed of three microchannels (with two inlets), a waste reservoir, and a circular central cavity where a cylindrical rotary valve was placed, connecting the microchannels and the waste reservoir. The cylindrical rotary valve consists of a hole at the top for inserting the sample and two parallel holes at the bottom. The cylindrical rotary valve is equipped with a handle to facilitate rotation and is conceived to direct the flow of the different solutions needed to actuate the different immunoassay steps. The microchannels’ detailed description is described below. Microchannel 1 (M1) is used to perform the washing steps in the immunoassay (see the *Tf Immunoisolation and Tf-Bound Iron Release* section). The washing buffer is introduced through its inlet, which is connected to the waste reservoir when the cylindrical rotary valve is open. Microchannels 2 (M2) and 3 (M3) are used to transport the sample from the immunoassay module to the 3D-sensor. The inlet of the M2, where the measurement buffer is introduced, connects the cylindrical rotary valve containing the sample. This cylindrical rotary valve, in turn, connects to the M3, where the sample is carried by the measurement buffer toward the 3D-sensors. Both microchannels, M2 and M3, were carefully designed with a 10° inclination to promote hydrodynamic-assisted capillary flow, thereby facilitating the efficient delivery of fluids to the cylindrical rotary valve (M2) and the interchangeable 3D-sensor (M3).

The 3D-sensor (Figure 1A, electrochemical cell) was printed in sequential steps using two types of filaments, i.e., PETG for the structural components (base and walls of the 3D-sensor) and PLA-CB for the conductive elements (contacts and electrodes). The printing process began with the PETG base layer. Once completed, the conductive PLA-CB filament was used to print the working, counter, and reference electrodes, along with their respective contacts, directly onto the base. Afterward, the three side walls that define the electrochemical cell chamber were printed. A wall opening was included to allow fluid to flow from the upstream immunoassay module to the sensing area. Eventually, inks were gently brushed to finalize the reference and working/counter electrodes, using Ag/AgCl and carbon ink, respectively. Then, a thermal curing step was undergone at 70 °C for 30 min. The working electrode was finally modified with 6 μL of the AuNP colloidal solution via drop casting.

Up to 64 disposable 3D-sensors can be printed in a single 3D printing mat, with a printing time of approximately 5 h and a cost of approximately €1.70/mat. As well it is also possible to print in series immunoassay modules and their accessories, according to the need.

An exploded view of magnet and rotatory valve holders as well as interchangeable 3D-sensor is shown in Figure 1B. The immunoassay module also features a cavity in its base with a cylindrical recess designed to accommodate a magnet, coinciding with the position of the working electrode from the electrochemical cell. A secondary cavity is situated at the rear of the platform, designed to accommodate a rectangular support with a cylindrical recess that accommodates another magnet, coinciding with the center of the cylindrical rotary valve, for use in the corresponding immunoassay step.

Video S1 shows the visualization of the on-device TSAT assessment using the 3D-EMD, where colored solutions were used exclusively for illustrative purposes.

### 3D-EMD-Based TSAT Assessment in Serum Samples

#### Tf Immunoisolation and Tf-Bound Iron Release

Anti-Tf-MB, complex formation procedure reported in the *Material* section, was resuspended in 50 μL of human serum sample (containing Tf) and placed into the central cavity of the cylindrical rotary valve, via the hole at the top, and incubated for 45 min at room temperature. At this stage, the valve is closed and does not allow flow along the microchannels.

Next, the rectangular support containing the magnet was inserted into the rear of the immunoassay module, aligning with the center of the cylindrical rotary valve, securing the Tf-anti-Tf-MBs complex. After 2 min, the supernatant was removed by moving the cylindrical rotary valve to connect the M1 to the waste reservoir. Two washing steps were performed, flushing 100 μL of PBS (0.1 M, pH 7.4) through the inlet of M1. Then, the cylindrical rotary valve was turned close, and the rectangular support containing the magnet was removed, allowing the release of the Tf-anti-Tf-MBs complex.

Tf-anti-Tf-MBs complex was resuspended in 50 μL of BR buffer (0.2 M, pH 3.0) and left to react for 15 min (the buffer was inserted into the circular central cavity via the hole at the top). The release of the iron from Tf was induced by the acidic medium. Eventually, the cylindrical rotary valve was turned to connect M2 and M3, and the solution was moved through air flushing in the M2 inlet toward the 3D-sensor, equipped with a magnet at the base of the working electrode. In this way, the Tf-anti-Tf-MBs complex was retained on the working electrode, and the Fe^3+^ remained free in solution. The electrochemical determination of Tf and Fe^3+^ was performed according to *Tf and Tf-Bound Iron Determination for TSAT Assessment* section.

#### Tf and Tf-Bound Iron Determination for TSAT Assessment

The simultaneous determination of Tf and Tf-bound iron at the 3D-sensor was performed via square wave voltammetry (SWV). Before measurement, a potential of −0.1 V was applied for 30 s to force the serum iron (Fe^3+^) at its reduced state (Fe^2+^). SWV was performed within an equilibration time of 5 s, in a potential range from −0.3 to +1.6 V, with a step potential of 0.005 V, an amplitude of 0.05 V, and a frequency of 2 Hz. Measurements were performed in triplicate using three independent 3D-EMDs. Control tests and blank samples (BR buffer) were subjected to the same analysis and testing protocol.

TSAT assessment in human serum samples was obtained by extrapolating the Tf and Tf-bound iron concentration from the respective calibration curves. Tf concentration (g L^–1^) was converted to TIBC, the maximum amount of iron that can be captured by Tf, using a conversion factor (×1.4) to obtain TIBC in μg mL^–1^; this conversion factor takes into account that each mol of Tf (average molecular weight of 79.5 kDa) can bind 2 mol of iron (molecular weight of 55.8 Da).^32^ Eventually, TSAT was calculated by dividing the concentration of Tf-bound iron by TIBC, according to the following formula

$$\mathrm{TSAT}\left(\%\right)=\frac{\left[\mathrm{Tf bound iron}\right]}{\left[\mathrm{TIBC}\right]}\times 100$$

## Results and Discussion

### 3D-EMD Design for Integrated TSAT Assessment in Serum Samples: Conceptual Integration and Assay Workflow

Figure 2 illustrates the conceptual integration of the assay workflow on board on the 3D-EMD for the simultaneous detection of Tf and Tf-bound iron (named Fe^3+^ for simplicity) for TSAT assessment in human serum samples, including cylindrical rotary valve position and flow direction schematics.

**Figure 2:**
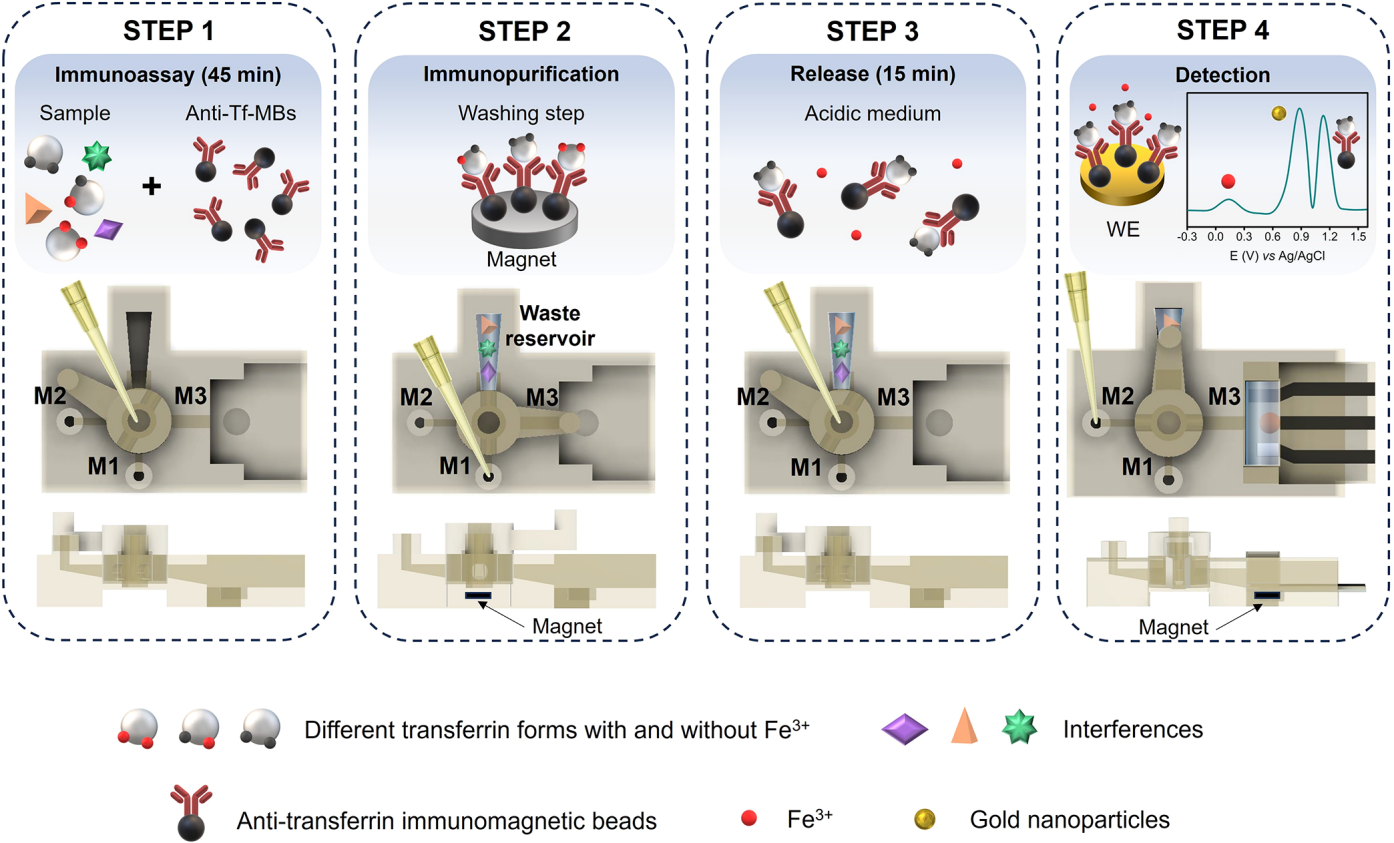
Conceptual integration of the assay workflow on board on the 3D-EMD for the simultaneous detection of Tf and Tf-bound iron (Fe^3+^) for TSAT assessment in human serum samples, including cylindrical rotary valve position and flow direction schematic.

*Step 1* – Immunoassay for selective Tf isolation. Anti-Tf-MBs are resuspended directly in human serum sample (containing Tf) into the circular central cavity of the cylindrical rotary valve. At this step, the cylindrical rotary valve is closed.

*Step 2* – Tf Immunopurification. The cylindrical rotary valve is turned to connect M1 to the waste reservoir. The excess of reagents is directed toward the waste reservoir by flushing the washing solution (PBS) into the M1 inlet. This eliminates potential contaminants and prevents cross-contamination, also eliminating unbound iron. At this step, the removable magnet is inserted under the device to retain the Tf-anti-Tf-MBs complex.

*Step 3* – Iron release. The cylindrical rotary valve is moved to the closing position, and the support containing the magnet is removed. BR buffer (0.2 M, pH 3.0) is introduced into the circular central cavity of the cylindrical rotary valve, resuspending the Tf-anti-Tf-MBs complex. The acidic pH allows the release of the bound iron from the Tf.

*Step 4* – Tf and Fe^3+^ simultaneous detection. The 3D-sensor is inserted into the 3D click holder of the immunoassay module, then the cylindrical rotary valve is turned to connect the M2 and M3. Then, with a micropipette, air was injected into the inlet of M2, transporting the sample from the circular central cavity to the electrochemical cell, which contained a magnet at the base of the working electrode. In this way, the Tf-anti-Tf-MBs complex is held back on the working electrode surface and the released Fe^3+^ (this was the iron tied to the Tf) remains free in the working solution. Eventually, the simultaneous electrochemical detection of the Tf and Fe^3+^ is performed by SWV after ultrafast electrochemical reduction of Fe^3+^ to Fe^2+^ (−0.1 V, 30 s). The SW-voltammogram was characterized by the presence of three main peaks, attributable to Fe^2+^ oxidation (around +0.15 V), AuNP-modified electrode oxidation (around +0.85 V), and Tf oxidation (around +1.17 V); this last peak is given by the accessible electroactive amino acids present in Tf as cysteine, tryptophan, and tyrosine.^39,40^

Specifically, the 3D-EMD was also designed to facilitate reagent and sample fluid movement by optimizing (i) the slope and microchannel dimensions, (ii) the inlet design and tip holder, and (iii) the cylindrical rotary valve configuration (see Figure S1 for details). (i) A slight inclination of 10° was introduced to promote both capillary-driven and gravity-assisted hydrodynamic flow, thereby avoiding reagent stagnation in the microchannels (see M3 in Figure S1 for details), while the channel dimensions were minimized to reduce reagent consumption; (ii) the channel inlets were designed to be compatible with micropipette tips for straightforward reagent and sample loading; and (iii) the cylindrical rotary valve was designed to prevent liquid leakage or cross-contamination while allowing air flow to efficiently drive liquid displacement through the channels.

### Tf and Tf-Bound Iron Simultaneous Electrochemical Sensing at the 3D-Interchangeable Sensor

At first, the 3D-sensor was optimized to allow the simultaneous determination of Tf and Tf-bound iron (Fe^3+^), since this is not possible with the pristine one. It is well-known that Tf is electroactive at carbon electrode surfaces, given the presence of electroactive amino acid residues,^39,40^ while iron itself results electrochemically inert. For this reason, the 3D-sensor was modified using different kinds of nanomaterials (NM). In particular, AuNPs and GQDs were tested given their well-known electrocatalytic ability.^41,42^ Thus, the determination of Fe^3+^ was attempted via SWV using 3D-sensors modified with AuNPs and GQDs (for simplicity, sensors were modified using the same NM volumes). Figure 3A demonstrates how both NM return an anodic peak centered around +0.15 V, not visible in their absence; in particular, the AuNPs allow for obtaining a significantly higher oxidation current.

**Figure 3:**
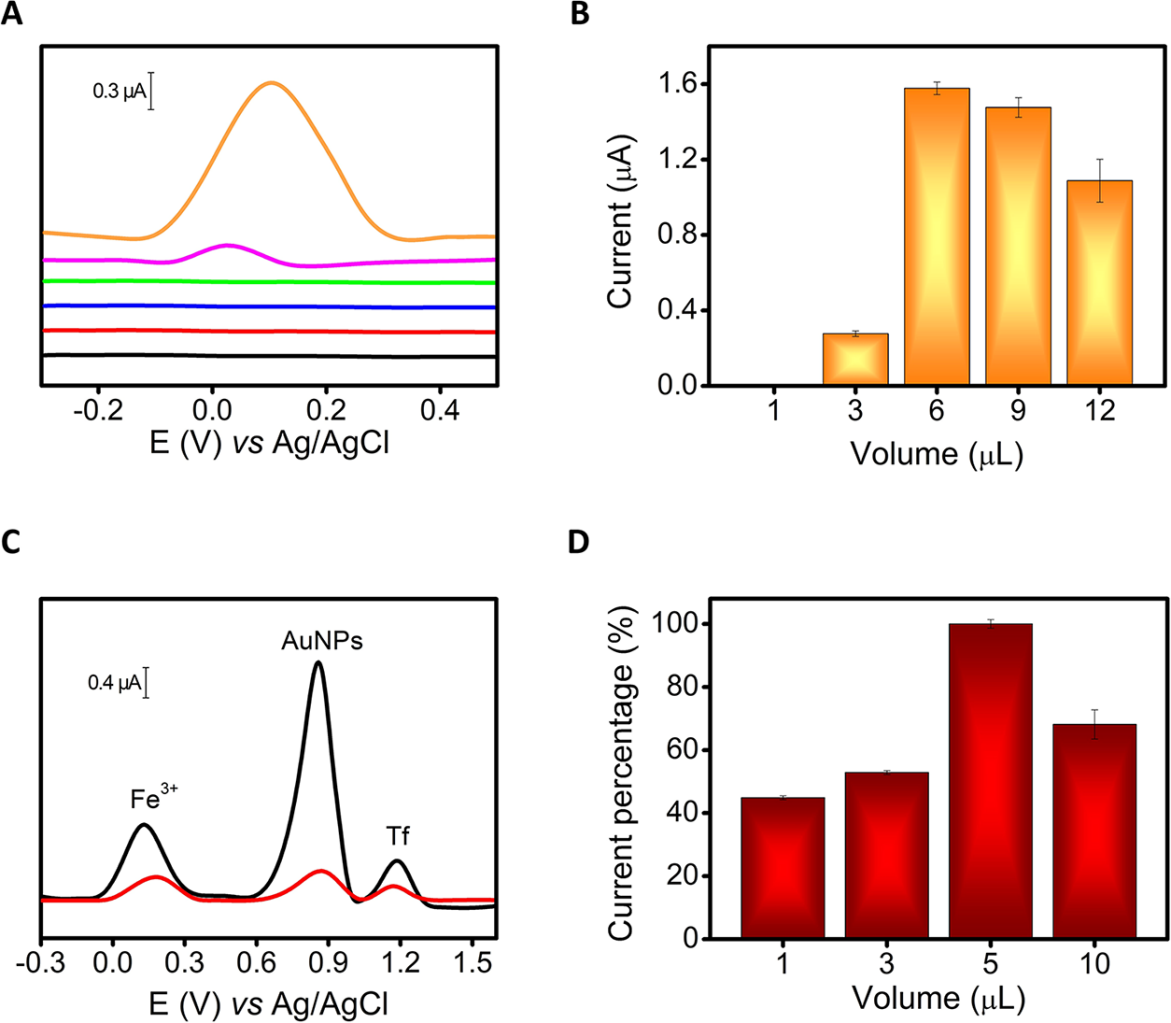
(A) Voltammograms obtained via SWV for 70 μg mL^–1^ Fe^3+^ in BR buffer at 3D-sensor with carbon ink only (green line), 3D-sensor ink-GQDs (purple line), and 3D-sensor ink-AuNPs (orange line). Voltammograms obtained via SWV as control (only BR buffer, without Fe^3+^) at 3D-sensor with carbon ink only (black line), 3D-sensor ink-GQDs (red line), and 3D-sensor ink-AuNPs (blue line). The “Fe^3+^ peak” is attributed to the oxidation of Fe^2+^, derived from the previous electrochemical reduction of the serum iron (Fe^3+^ + 1e^–^ → Fe^2+^), carried out applying a potential of −0.1 V for 30 s. (B) Average peak intensities obtained via SWV analyzing 100 μg mL^–1^ Fe^3+^ in BR buffer at 3D-sensor with carbon ink modified with increasing amount of AuNPs. (C) Voltammograms obtained for 0.5 g L^–1^ Tf and 70 μg mL^–1^ Fe^3+^ in BR buffer at 3D-EMD using SWV (black line) and DPV (red line). (D) Normalized current intensity for anodic peak obtained via SWV testing different amounts of MBs to capture 3 g L^–1^ Tf in BR buffer following the 3D-EMD analytical procedure; the currents were normalized for the higher current value obtained. SWV parameters: equilibration time 5 s, start potential −0.3 V, end potential +1.6 V, frequency 2 Hz, amplitude 0.05 V, and step potential 0.005 V; the background signal was linearized. DPV parameters: equilibration time 5 s, start potential −0.3 V, end potential +1.6 V, modulation time 0.5 s, amplitude 0.05 V, and step potential 0.005 V; the background signal was linearized. Error bars represent the mean values ± standard deviation (*n* = 3).

Control measurements performed produced negligible electrochemical signals, attributing the sensing ability to the employed NM. Based on these results, AuNPs were selected as NM for further experiments. The amount of the AuNPs was thus optimized to find the best compromise between analytical signal and background noise. Figure 3B proves how 6 μL of AuNP colloid allows to obtain the best Fe^3+^ detection, and for this reason, it was selected as the optimum volume for the following experiments. To better understand the AuNP contribution, the electrochemical features of the 3D-sensors were investigated via cyclic voltammetry (CV), using 5 mM K_4_Fe­(CN)_6_/K_3_Fe­(CN)_6_ in 0.1 M KCl as inner-sphere redox probe. Using the same probe, the minimal working volume needed to return reliable output in the 3D-EMD was studied to ensure the correct workflow, with the idea to find a compromise between lower dilution of the sample and higher signal intensity (see SI for details). From the electrochemical experiments shown in Figure S3, the role of AuNPs in enhancing the sensitivity of the electrochemical detection was confirmed, as well as the minimum volume required to provide a reliable and reproducible signal, which was 50 μL.

Afterward, the simultaneous detection of Tf and Tf-bound iron was attempted using different electroanalytical techniques; in particular, SWV was compared with differential pulse voltammetry (DPV). Figure 3C demonstrates how both SWV and DPV allow the detection of the two analytes simultaneously, allowing a peak resolution between Fe^3+^ (∼ +0.15 V) and Tf (∼ +1.17 V). The SWV obtained peaks appeared more defined and significantly higher (2.7-fold for Fe^3+^ and 3.1-fold Tf) compared to the ones obtained via DPV; therefore, SWV was selected. Eventually, the MB volume used to selectively capture of Tf from serum samples in the immunoassay step was optimized. Different volumes of MBs were studied in the presence of Tf via SWV; recording the intensity of the voltametric peak obtained around +1.17 V (for details, see the *Tf Immunoisolation and Tf-Bound Iron Release* section*)*. Figure 3D represents the normalized current intensity obtained depending on the MB volume. An increase in the intensity of the anodic peak was obtained according to the MB volume, reaching its maximum value with 5 μL of MBs (10 mg mL^–1^); thus, this MB amount was selected for the following experiments.

### 3D-EMD Analytical Performance and Serum Samples Analysis from Ischemic Stroke Patients

The analytical performance of the 3D-EMD-based Tf and Tf-bound iron determination was then studied. Linear calibration plots were obtained independently for the two analytes. Each point on the calibration plots was obtained using three independent disposable 3D-sensors. The main characteristics of the methodological calibration are summarized in Table 1.

**Table 1:** Analytical Characteristics of Calibration Curve for Tf Using External Standard Method and Fe^3+^ Using Standard Additions in Certified Human Serum (*n* = 3)

analyte	linear range	*R* ^2^	a ± s_a_ (μA)	b ± s_b_	RSD_Slope_ (%)	LOD
Tf	1–10 g L^–1^	0.998	1.09 ± 0.03	0.218 ± 0.005 (μA g^–1^ L)	2	0.4 g L^–1^
Fe^3+^	0–100^a^ μg mL^–1^	0.990	0.1 ± 0.3	0.085 ± 0.005 (μA μg^–1^ mL)	6	0.6 μg mL^–1^

a Added Fe^3+^ concentration.

Figure 4A reports the voltammograms obtained for Tf at different concentrations, recording the voltammetric peak intensity around +1.17 V. The external calibration plot resulted linear between 1–10 g L^–1^, yielding an excellent determination coefficient (*R* ^2^ = 0.998). An excellent reproducibility was obtained comparing three different independent calibration plots (RSD_slope_ = 2%). The limit of detection (LOD) obtained was 0.4 g L^–1^ (calculated as 3 S/N criteria, using the standard deviation of the intercept). Considering that the Tf concentration in human serum samples ranged between 2 and 3 g L^–1^,^43^ the system is potentially exploitable for Tf sensing.

**Figure 4:**
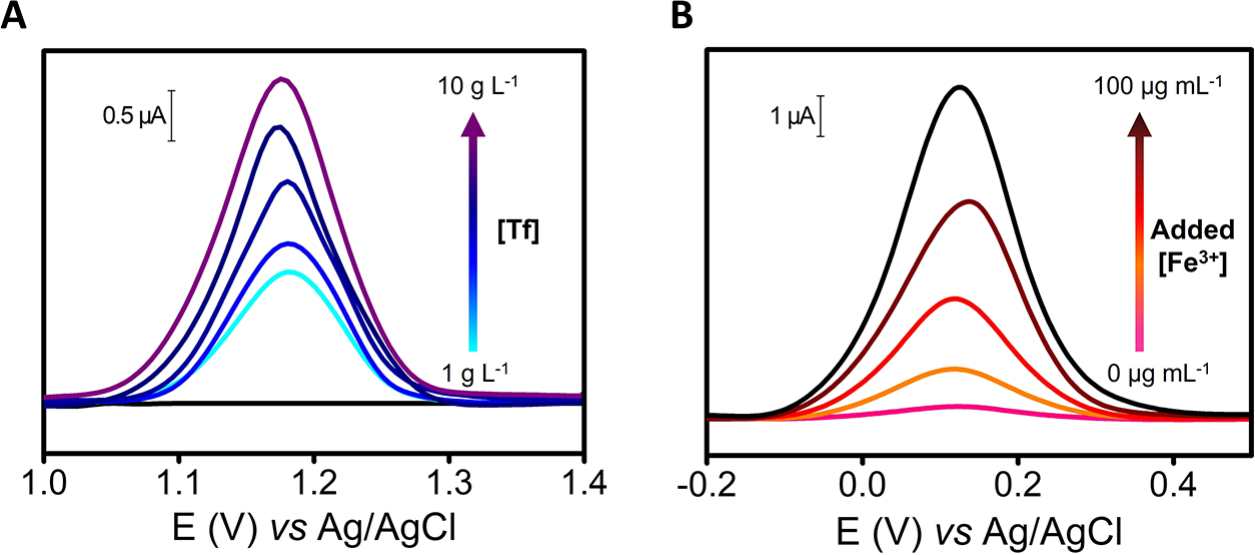
(A) Voltammograms corresponding to Tf standard solutions (in BR solution) from 1 to 10 g L^–1^ using the 3D-EMD. (B) Voltammograms corresponding to Fe^3+^ standard additions in certified human serum (in BR solution) from 0 to 100 μg mL^–1^ additions of Fe^3+^ using the 3D-EMD. SWV parameters: equilibration time 5 s, start potential −0.3 V, end potential +1.6 V, frequency 2 Hz, amplitude 0.05 V, and step potential 0.005 V; the background signal was linearized.

Figure 4B shows the voltammograms obtained for Tf-bound iron (Fe^3+^) using a calibration standard addition in certified human serum, constructed recording the voltammetry peak current at +0.15 V. Additions of 0, 20, 40, 70, and 100 μg mL^–1^ of Fe^3+^ were performed. This approach was necessary to correct the matrix effects observed in serum, which significantly enhanced the electrochemical signal of Fe^3+^ compared to calibrations performed in BR buffer. The linear part of the plot resulted linear up to 100 μg mL^–1^ additions of Fe^3+^, yielding an excellent determination coefficient (*R* ^2^ = 0.990). A good reproducibility was obtained comparing three different independent calibration plots (RSD_slope_ = 6%), and a LOD of 0.6 μg mL^–1^ was obtained. The system result suitable for iron analysis in human serum since the normal range reported in the literature is enclosed between 0.7 and 1.7 μg mL^–1^.^44^ Notably, these results were obtained without any sample preconcentration, as Fe^3+^ was electrochemically reduced *in situ* with enough sensitivity and without the use of chemical reducing agents, demonstrating the reagent-free operation of the developed device. Also, interestingly, given the excellent robustness of the method (RSD_slope_ ≤ 6%) for the determination of both biomarkers constituting TSAT, a simplified one-point calibration approach could be envisioned in the future, which could also facilitate its implementation for POCT.

Table 2 reports the data obtained for the determination of Tf and Tf-bound iron in certified reference material (human serum), subjected to the complete analytical procedure based on the 3D-EMD. The data obtained demonstrates the ability of the proposed device to ensure an accurate and reproducible simultaneous determination of the two analytes.

**Table 2:** Analysis of Certified Reference Material using the 3D-EMD (*n* = 3)

		3D-EMD value		
analyte	certified value	*x̅* ± *s*	RSD (%)	*E*_r_ (%)
serum iron	1.15 μg mL^–1^	1.09 ± 0.05 μg mL^–1^	4	5
Tf	1.96 g L^–1^	1.92 ± 0.05 g L^–1^	3	2

Eventually, the 3D-EMD has also been tested for the simultaneous assessment of TSAT in 5 pseudonymized serum samples from ischemic stroke patients of the TANDEM-1 study (see the *Reagent and Sample* section). The samples were analyzed in parallel using the 3D-EMD and the reference method, i.e., the urea-PAGE.^35^ Figure 5A reports the correlation plot obtained, and Table S1 lists the obtained data. Figure 5B reports a voltammogram obtained from sample S2 as an example (red line), where the simultaneous detection of Tf-bound iron (Fe^3+^) and Tf can be observed.

**Figure 5:**
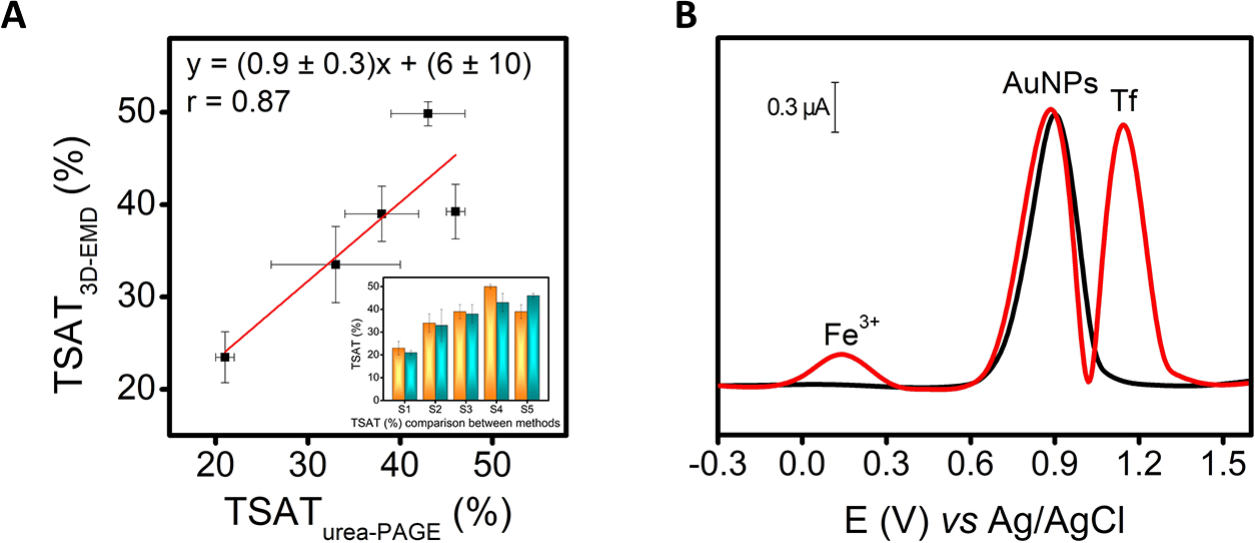
(A) Correlation plot obtained analyzing TSAT via 3D-EMD and urea-PAGE.^35^ Inset: Data comparison for TSAT values obtained with 3D-EMD (orange bar) and urea-PAGE method^35^ (cyan bar). (B) Voltammograms obtained via SWV analyzing sample S2 using the 3D-EMD (red line); black line indicates the measure obtained in only 0.2 M BR buffer (pH 3.0). The “Fe^3+^ peak” is attributed to the oxidation of Fe^2+^, derived from the previous electrochemical reduction of the serum iron (Fe^3+^ + 1e^–^ → Fe^2+^), carried out applying a potential of −0.1 V for 30 s. SWV parameters: equilibration time 5 s, start potential −0.3 V, end potential +1.6 V, frequency 2 Hz, amplitude 0.05 V, and step potential 0.005 V; the background signal was linearized. Error bars represent the mean values ± standard deviation (*n* = 3).

A good correlation was obtained between the two methods (r = 0.87). More importantly, no significant numerical differences were found, as demonstrated by the value obtained for the slope (0.9 ± 0.3) and the intercept (6 ± 10), which included 1 and 0, respectively (*p* < 0.05). These findings are particularly noteworthy since the urea-PAGE method is a free-interference method, and consequently, accuracy was demonstrated.

Furthermore, this excellent correlation exists not only with the urea-PAGE method, but also with methods previously proposed by our group, based on a colorimetric paper-based analytical device^29^ and a dual colorimetric-electrochemical microfluidic paper-based analytical device,^30^ showing significant similarities in the analysis of the same samples. It should be noted that the values derived from the 3D-EMD are closer to the reference values (urea-PAGE), compared to those obtained in previous studies. This suggests that the simultaneous electrochemical determination of Fe^3+^ and Tf, for the first time proposed in this device, enhances the accuracy of the method, probably due to the enhanced precision offered by electrochemical detection compared to the visual/colorimetric one. Moreover, the use of the same electrochemical detection principle simplifies, avoids the use of reductive/colorimetric chemistry for iron determination and enables the direct analysis of serum samples due to its insensitivity to sample coloration as well the integration of all stages, representing a significant step forward in the development of a *point-of-care* device for ischemia.

On the other hand, although the previously proposed paper-based analytical devices^29,30^ have shown to be a highly competitive and affordable compared to the conventional urea-PAGE method, they have the main drawback of the need to perform the immunoassay and immunopurification steps outside the device. It inherently adds additional complexity, limiting its applicability in decentralized analysis. In turn, urea-PAGE requires expensive equipment and needs to be performed in clinical laboratories or equipped medical centers with trained personnel.^45^ For the sake of clarity, it should be noted that urea-PAGE has the advantage of separating and detecting the different forms of Tf (apo-Tf, monoferric-Tf, and diferric-Tf), however, this requires long time (more than 18 h).

In this context, importantly, 3D-EMDs-based approach takes ∼60 min (since the synthesis of anti-Tf-MBs can be offered as a kit to add to the device) and has the advantage of being unaffected by the color of the sample, thanks to the use of anti-Tf-MBs and the electrochemical detection; therefore, serum, blood, or hemolyzed samples may be potentially analyzed. To the best of our knowledge, the 3D-EMD results in a pioneering device for measuring TSAT, making it a potential candidate as POCT, since it meets important features, such as being affordable, sensitive, specific, portable, easy-to-use (usable by healthcare workers), and deliverable to end-users, as well as requiring a very low clinical sample amount (50 μL), even requiring a sample volume two to three times lower than that used in previous paper-based analytical approaches.

Eventually, the stability of the interchangeable electrochemical sensors and the anti-Tf-MBs complex were also studied. To this aim, 3D-sensors were stored at room temperature, protected from light, in an unmodified atmosphere, and every 5/7 days tested toward K_4_Fe­(CN)_6_/K_3_Fe­(CN)_6_, recording the anodic peak intensity obtained via CV. Figure S4A demonstrates that, up to 17 days, there are no significant differences in the electrochemical signal obtained (*t* test, α = 0.05, two-sided, n = 3). Anti-Tf-MBs complex was stored at 4 °C in PBS protected from light. The stability was investigated by analyzing their binding efficiency and signal response toward Tf every 5/7 days. Figure S4B reports the normalized current intensity obtained. Along 20 days, there were no significant differences in current intensity (*t* test, α = 0.05, two-sided, n = 3). Overall, both 3D-EMD sensors and the immunoassay demonstrated satisfactory stability under the tested conditions, ensuring reliable performance over around 20 days. The stability obtained can be improved by working on the storage conditions in the case of sensors, while the anti-Tf-MBs complex can be prepared directly *in situ* on the cylindrical rotary valve before the immunoassay step, avoiding storage limitations.

## Conclusions

A 3D-printed integrated device for TSAT assessment in human serum samples from ischemic stroke patients was successfully designed, developed and validated. The device incorporates an immunoassay module for selective Tf capture and hosts interchangeable 3D-sensors able to determine Tf and Tf-bound iron simultaneously. The device integrates the entire analytical procedure in a single compact system, reducing sample handling, analysis time, and the risk of contamination, while requiring only 50 μL of clinical sample. The 3D-EMD demonstrated a sensitivity suitable for TSAT assessment in serum, along with precision (RSD ≤ 4%) and accuracy (*E*_r_ ≤ 5%), validated using both certified reference material and clinical samples from ischemic stroke patients. Data obtained with the 3D-EMD showed an excellent correlation with the urea-PAGE reference method, allowing a drastic reduction of the analysis time (60 min *vs* 18 h). The modular and reusable design of the 3D-EMD, equipped with interchangeable 3D-printed sensors, results cost-effective (∼0.4 € per device), and all the device components can be produced automatically in series, even they can be envisioned as a cost-effective disposable device for routine use in *point-of-care* settings. Also, due to its portability, rapid workflow, minimal reagent consumption, and insensitivity to sample coloration, unlike colorimetric detection, the 3D-EMD offers a promising POCT solution for TSAT assessment, enabling the direct analysis of serum, blood, or even hemolyzed samples and supporting clinical decision-making in time-critical conditions such as ischemic stroke. Future work will explore the further automation of the workflow will be attempted to make it increasingly closer to the concept of a POCT device. Beyond that, this work highlights the tremendous potential of 3D printing as a versatile technology for the design and development of (micro)­fluidic systems, with relevance in the context of diagnostic devices. In this regard, the design flexibility provided by 3D printing readily enables the incorporation of multiple channels or electrochemical cells within a single device, facilitating the development of multiplexed platforms for simultaneous biomarker detection. Likewise, the manually operated rotary valve currently used for fluid control could be easily adapted for motorized or microcontroller-based actuation, paving the way toward a fully automated and user-friendly system.

## Supplementary Material





## Notes

Abbreviations; supporting sections: 3D-components details; 3D-sensors cyclic voltammetry study and working volume optimization; supporting figures and tables; Figure S1: 3D-EMD components exploded with the respective geometries and dimensions; Figure S2: Photo of the complete 3D-EMD; Figure S3: Cyclic voltammetry study and working volume optimization; Table S1: Comparison of TSAT values in serum samples from ischemic stroke patients obtained by 3D-EMD and by urea-PAGE; Figure S4: 3D-sensor and anti-Tf-MBs stability studies (PDF)
Visualization of the on-device TSAT assessment via 3D-EMD (AVI)

## Abbreviations

3D: three-dimensional
3D-EMD: three-dimensional-printed electrochemical microfluidic device
Anti-Tf: anti-transferrin antibody
Anti-Tf-MBs: anti-transferrin immunomagnetic beads
AuNPs: gold nanoparticles
BR: Britton–Robinson
CB: carbon black
CV: cyclic voltammetry
DPV: differential pulse voltammetry
ECSA: electrochemical active surface area
EMD: electrochemical microfluidic device
FDM: fused deposition modeling
FFF: fused filament fabrication
LOD: limit of detection
M1: microchannel 1
M2: microchannel 2
M3: microchannel 3
MBs: immunomagnetic beads
NM: nanomaterials
PBS: phosphate buffer saline
PETG: polyethylene terephthalate glycol
PLA: polylactic acid
POCT: *point-of-care* testing
RSD: relative standard deviation
SWV: square wave voltammetry
Tf: transferrin
TIBC: total iron-binding capacity
TSAT: transferrin saturation percentage
urea-PAGE: urea polyacrylamide gel electrophoresis
UV–vis: ultraviolet–visible
