# Supplementary material for: Integrated 3D-Printed Microfluidic Device for Immunocapture and Electrochemical Assessment of Transferrin Saturation in Point-of-Care Stroke Diagnostics
Source: ACS Sens. 2025 Dec 26;11(1):237–46. doi: 10.1021/acssensors.5c02834 (PMC12836336; doi:10.1021/acssensors.5c02834)
Supplement: Supplementary file 1 [file se5c02834_si_001.pdf]

SUPPORTING INFORMATION

Integrated 3D-Printed Microfluidic Device for Immunocapture and Electrochemical Assessment of Transferrin Saturation in *Point-of-Care* Stroke Diagnostics

Davide Paolini[a,b,†], Silvia Dortez[a,†], Marta Pacheco[a], Teresa Gasull[c], Dario Compagnone[b], Flavio Della Pelle[b], Alberto Escarpa[a,d*]**

[a] Department of Analytical Chemistry, Physical Chemistry and Chemical Engineering, University of Alcala, 28805, Alcala de Henares, Madrid, Spain.

[b] Department of Bioscience and Technology for Food, Agriculture and Environment, University of Teramo, Campus “Aurelio Saliceti” Via R. Balzarini 1, 64100, Teramo, Italy.

[c] Cellular and Molecular Neurobiology Research Group, Department of Neurosciences, Germans Trias i Pujol Research Institute (IGTP), 08916, Badalona, Barcelona, Spain.

[d] Chemical Research Institute “Andrés M. Del Río” (IQAR), University of Alcala, 28805, Alcala de Henares, Madrid, Spain.

Corresponding authors:

*Alberto Escarpa, Department of Analytical Chemistry, Physical Chemistry and Chemical Engineering and Chemical Research Institute “Andres M. Del Rio”, Universidad de Alcalá, Madrid, Spain (alberto.escarpa@uah.es)

**Flavio Della Pelle, Department of Bioscience and Technology for Food, Agriculture and Environment, University of Teramo, Campus “Aurelio Saliceti” Via R. Balzarini 1, 64100, Teramo, Italy (fdellapelle@unite.it).

[†] D.P. and S.D. contributed equally to this paper

ORCID number:

Davide Paolini: 0009-0002-8774-6464

Silvia Dortez: 0000-0002-1999-5605

Marta Pacheco: 0000-0001-9546-9108

Teresa Gasull: 0000-0002-9321-1741

Dario Compagnone: 0000-0001-7849-8943

Flavio Della Pelle: 0000-0002-8877-7580

Alberto Escarpa: 0000-0002-7302-0948

TABLE OF CONTENTS

	Pg.
**ABBREVIATIONS**	S3
**SUPPORTING SECTIONS**	
3D-components details	S4
3D-sensors cyclic voltammetry study and working volume optimization	S5
**SUPPORTING FIGURES AND TABLES**	
**Figure S1.** 3D-EMD components exploded with the respective geometries and dimensions	S6
**Figure S2.** Photo of the complete 3D-EMD	S7
**Figure S3.** Cyclic voltammetry study and working volume optimization	S8
**Table S1.** Comparison of TSAT values in serum samples from ischemic stroke patients obtained by 3D-EMD and by urea-PAGE	S9
**Figure S4.** 3D-sensor and anti-Tf-MBs stability studies	S10

ABBREVIATIONS

3D, three-dimensional; **3D-EMD**, three-dimensional-printed microfluidic device; **Anti-Tf**, anti-transferrin antibody; **Anti-Tf-MBs**, anti-transferrin immunomagnetic beads; **AuNPs**, gold nanoparticles; **BR**, Britton–Robinson; **CB**, carbon black; **CV**, cyclic voltammetry; **DPV**, differential pulse voltammetry; **ECSA**, electrochemical active surface area; **EMD**, electrochemical microfluidic device; **FDM**, fused deposition modelling; **FFF**, fused filament fabrication; **LOD**, limit of detection; **M1,** microchannel 1; **M2,** microchannel 2; **M3,** microchannel 3; **MBs**, immunomagnetic beads; **NM**, nanomaterials; **PBS**, phosphate buffer saline; **PETG**, polyethylene terephthalate glycol; **PLA**, polylactic acid; **POCT**, *point-of-care* testing; **RSD**, relative standard deviation; **SWV**, square wave voltammetry; **Tf**, transferrin; **TIBC**, total iron-binding capacity; **TSAT**, transferrin saturation percentage; **urea-PAGE**, urea polyacrylamide gel electrophoresis; **UV–vis**, ultraviolet–visible.

SUPPORTING SECTIONS

3D-components details

Figure S1 depicts the 3D-EMD components exploded with the respective geometries and dimensions; below is a detailed description of the same.

Immunoassay module. This module consisted of a rectangle (41 mm length x 22 mm width x 8 mm height), with a circular central cavity (10 mm diameter x 4 mm depth), a waste reservoir (12 mm length x 2 mm width at the point of intersection with the cylindrical rotary valve and 4 mm width at the end x 4 mm depth), and three microchannels (M1, M2, and M3) extending from the central cylindrical rotary valve. M1 possessed dimensions of 5 mm length x 2 mm width x 2 mm depth, with a 10° inclination towards the cylindrical rotary valve to facilitate hydrodynamic-assisted capillary flow. This microchannel also included a hollow cylindrical inlet with an external diameter of 4 mm, an internal diameter of 1 mm, and a height above the platform of 3 mm. M2 possessed dimensions of 7 mm length x 2 mm width x 2 mm depth, with a 10° inclination towards the cylindrical rotary valve. This microchannel also included a hollow cylindrical inlet with an external diameter of 4 mm, an internal diameter of 1 mm, and a height above the platform of 3 mm. M3 possessed dimensions of 6 mm length x 3 mm width x 2 mm depth, with a 10° inclination towards the electrochemical sensor to promote both capillary-driven and gravity-assisted hydrodynamic flow.
The immunoassay module also features a cavity in its base for the placement of the electrochemical sensor (15 mm length x 18 mm width x 5 mm depth). It has a cylindrical recess inside (4 mm diameter x 1.6 mm depth) designed to accommodate a magnet, which coincides with the position of the working electrode.
A secondary rectangular cavity (19 mm length x 11 mm width x 3 mm depth) is situated at the rear of the immunoassay module, intended to accommodate the rectangular support containing the magnet (18.5 mm length x 10.5 mm width x 2.5 mm depth).

Cylindrical rotary valve. The valve has a diameter of 10 mm, a height of 9 mm, two parallel holes at the bottom of 2.5 mm in diameter, and a hole at the top of 3.5 mm in diameter. The handle of the cylindrical rotary valve has dimensions of 10 mm length x 6 mm width x 3 mm depth.

Rectangular support. This support features a cylindrical recess (4 mm diameter x 1.6 mm depth), which functions as a holder for the magnet used in the immunoassay and coincides with the center of the cylindrical rotary valve.

Electrode support. The electrode support consisted of a rectangle 41 mm x 32 mm x 1 mm (length x width x thickness) where the three-electrode system was 3D-printed. This electrochemical cell consisted of three 16 mm x 2 mm x 0.4 mm (length x width x thickness) rectangles with a pitch separation of 5 mm in a microband configuration that was used for electrical connections, the reference electrode that was a rectangle of 3 mm x 2 mm x 0.4 mm (length x width x thickness), the working electrode that was a circle of 3 mm diameter, and the counter electrode that was a rectangle of 4 mm x 2 mm x 0.4 mm (length x width x thickness). The wall that delimits the working area of the electrochemical cell has dimensions of 6 mm x 13 mm x 5 mm (length x width x height), and the hole in the wall to allow fluid flow from the immunoassay module to the sensor has dimensions of 2 mm x 3.5 mm x 0.5 mm (length x width x thickness).

Figure S2 shows a photo of the 3D-EMD.

3D-sensors cyclic voltammetry study and working volume optimization

To better understand the gold nanoparticles (AuNPs) contribution, the electrochemical features of the 3D-sensors were investigated via cyclic voltammetry (CV), using 5 mM $K_4Fe(CN)_6/K_3Fe(CN)_6$ in 0.1 M KCl as inner-sphere redox probe. **Figure S3A** shows the cyclic voltammograms obtained in the presence and absence of AuNPs. The AuNPs presence induces a slight improvement of the peak intensity and peak-to-peak separation (i_{pa} = 61 ± 5 μA; ΔE = 361 ± 17 mV), compared to the unmodified sensor (i_{pa} = 59 ± 3 μA; ΔE = 402 ± 23 mV). This trend is confirmed by the electrochemical active surface area (ECSA) increasing observed in the presence of the AuNPs (0.132 ± 0.005 cm^2), higher compared to the unmodified sensor (0.102 ± 0.003 cm^2); ECSA was extrapolated according to Randles−Sevcik's theory, quasi-reversible systems under diffusion-controlled conditions. Considering the results obtained, we can state that AuNPs do not significantly improve the sensor's overall electrochemical characteristics. On the contrary, the presence of AuNPs is crucial for the detection of Tf-bound iron (Fe^{3+}), which in their absence is completely undetectable.

The optimal working volume of the 3D-printed electrochemical cell was studied to ensure the 3D-EMD correct workflow. Different volumes (20, 30, 50, and 100 μL) of $K_4Fe(CN)_6/K_3Fe(CN)_6$ were studied via CV. **Figure S3B** demonstrates how 50 μL of solution results in the best compromise between lower volume (lower dilution of the sample) and higher signal intensity.

SUPPORTING FIGURES AND TABLES

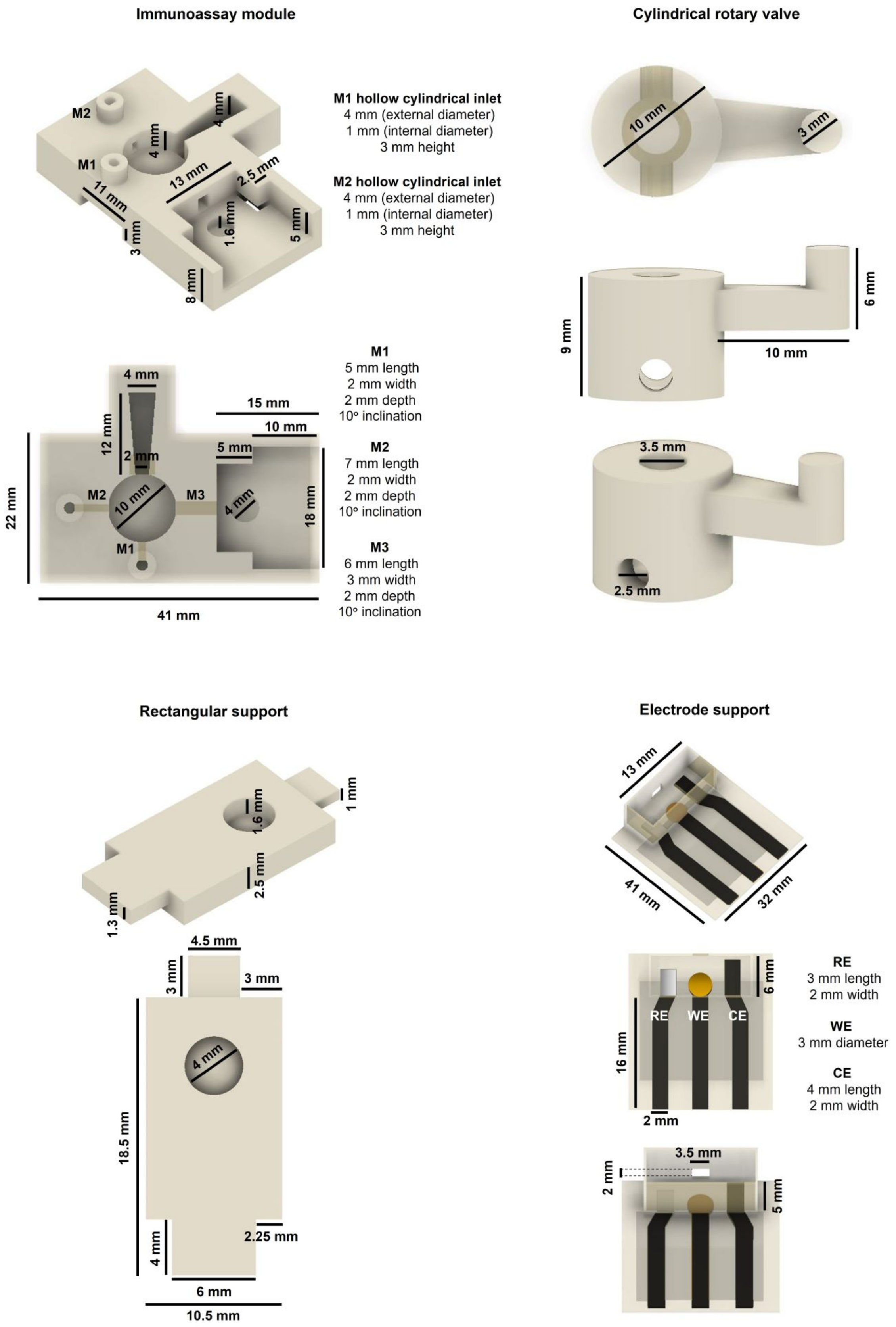

Figure S1. 3D-EMD components' detailed geometries and dimensions.

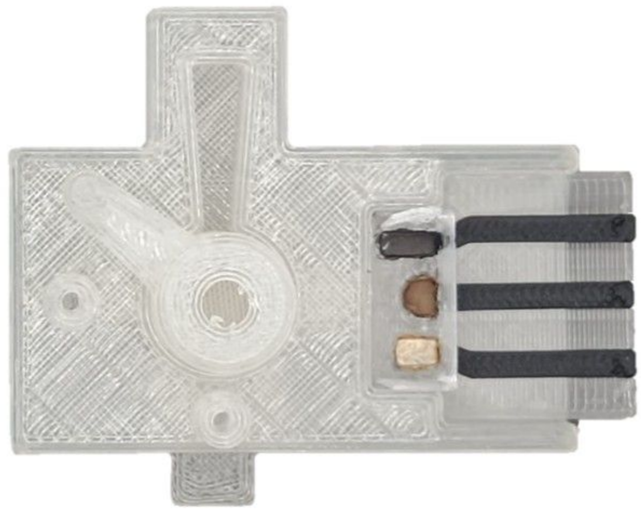

Figure S2. Photo of the complete 3D-EMD.

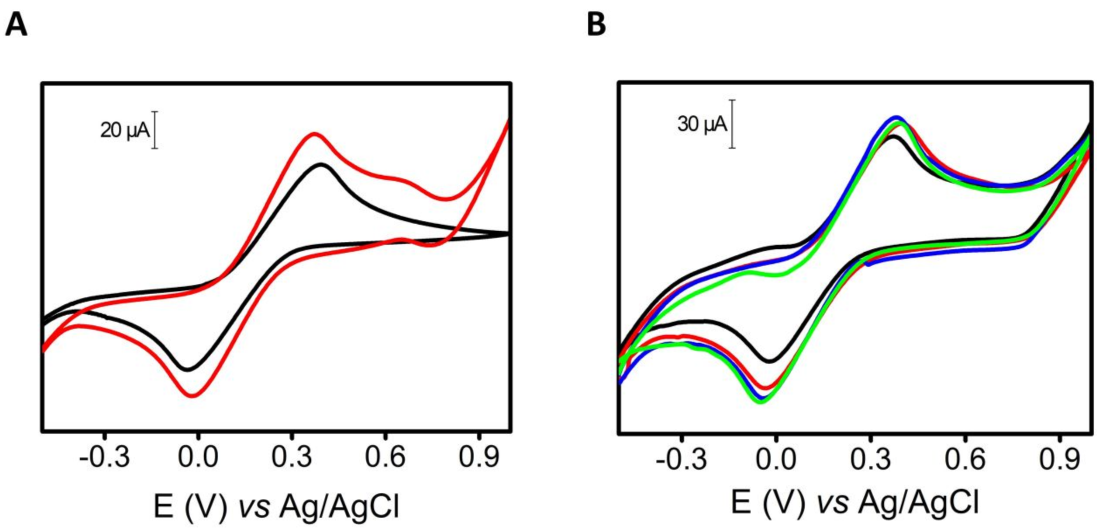

Figure S3. (A) CV of 5 mM $K_4Fe(CN)_6/K_3Fe(CN)_6$ in 0.1 M KCl obtained at 3D-EMD with carbon ink only (black line) and at 3D-EMD with carbon ink-AuNPs (red line). **(B)** CV obtained at 3D-EMD, analyzing different volumes of 5 mM $K_4Fe(CN)_6/K_3Fe(CN)_6$ in 0.1 M KCl: 20 µL (black line), 30 µL (red line), 50 µL (blue line), and 100 µL (green line). CV parameters: start potential –0.5 V, end potential +1.0 V, scan rate 0.025 V s^{-1} (n = 3).

Table S1. TSAT values in serum samples from ischemic stroke patients obtained by 3D-EMD and by urea-PAGE (n = 3).

Serum sample	$TSAT_{3D\text{-}EMD}$ (%)	$TSAT_{urea\text{-}PAGE}$ (%)
**S1**	23 ± 3	21 ± 1
**S2**	34 ± 4	33 ± 7
**S3**	39 ± 3	38 ± 4
**S4**	40 ± 3	50 ± 1
**S5**	39 ± 3	46 ± 1

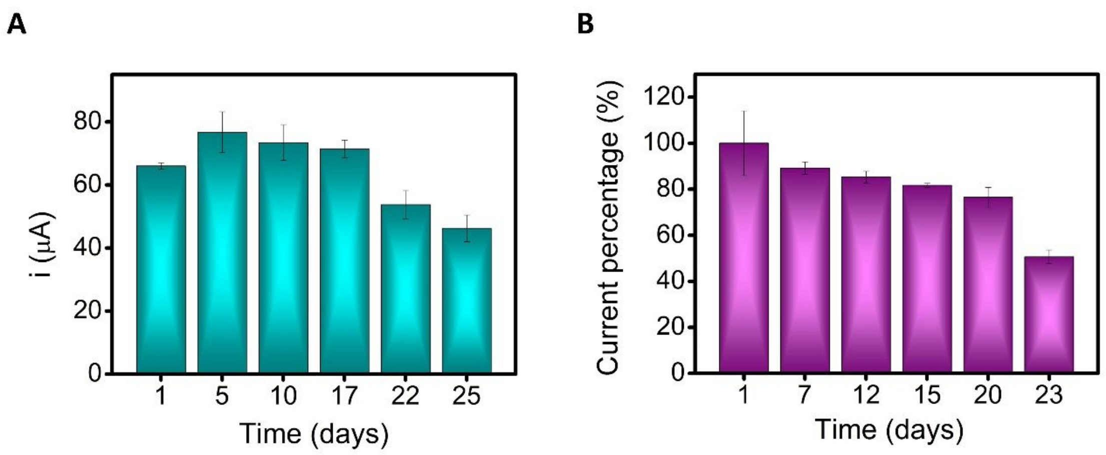

Figure S4. (A) Peak intensities obtained via CV of 5 mM $K_4Fe(CN)_6/K_3Fe(CN)_6$ in 0.1 M KCl for complete 3D-EMD at different storage times. **(B)** Normalized current intensity for anodic peak obtained by analyzing 3 g L^{-1} Tf solution via SWV testing anti-Tf-MBs at different storage times; the currents were normalized for the highest current value obtained. CV parameters: start potential –0.5 V, end potential +1.0 V, scan rate 0.025 V s^{-1}. SWV parameters: equilibration time 5 s, start potential −0.3 V, end potential +1.6 V, frequency 2 Hz, amplitude 0.05 V, and step potential 0.005 V. Error bars represent the mean values ± standard deviation (n = 3).
